# Green areas, clean air and cardiovascular health in the city of São Paulo: Áreas verdes, ar puro e saúde cardiovascular na cidade de São Paulo

**DOI:** 10.1590/1516-3180.2017.1351291216

**Published:** 2017-01-05

**Authors:** Paulo Andrade Lotufo

**Affiliations:** I MD, DrPH. Full Professor, Department of Internal Medicine, Faculdade de Medicina da Universidade de São Paulo (FMUSP), São Paulo (SP), Brazil.

This year of 2017 is the inaugural term for most mayors in Brazilian cities, including Mr. João Doria, in the largest city of Brazil, São Paulo. During the electoral period, debate on healthcare issues focused on access, either to medical care or to high-cost examinations. Mr. Doria prioritized access at primary healthcare units, so that people could undergo imaging examinations during the night with the claim of “no queues for examinations”! Mr. Doria’s proposal was driven by marketers and not by a serious evaluation of health determinants.

Considering that cardiovascular diseases are the leading cause of death in São Paulo, and most frequently among people living in the poorest districts,^1^^,^^2^ we have a question: How will Mr. Doria remedy the high and unequal burden of cardiovascular diseases? Will this be achieved through greater access to echocardiography, angiography, nuclear medicine etc.?

Absolutely not. This goal is more likely to be achieved through actions to improve and support cardiovascular health outside of the Health Department. Specifically, the mayor should look towards the Parks & Green Areas and Transportation Departments. We have enough evidence to advocate that increasing the green areas of the city and exchanging diesel-fueled buses for vehicles equipped with cleaner engines will have an impact on the burden of heart diseases and stroke that will benefit all the citizens of this city.

Our proposal is not presumptuous. Rather, it results from knowledge coming from contemporary research on cardiovascular epidemiology, including data from the Brazilian Longitudinal Study of Adult Health (ELSA-Brasil).^3^ The focus within cardiological preventive actions is shifting from only identifying lifestyle and genetic factors that predispose towards high incidence and lethality of cardiovascular diseases, to a more open view of the meaning of risk factors for a population, and not just for individuals.^4^ Air pollution, noise and physical inactivity may be consequences of the geography of cities, with long distances from home to work that place strain on accessing education, shopping and leisure activities.^5^

## DATA FROM THE BRAZILIAN LONGITUDINAL STUDY OF ADULT HEALTH (ELSA-BRASIL) 

ELSA-Brasil is a cohort of 15,105 men and women living in six large cities of Brazil: São Paulo, Rio de Janeiro, Salvador, Belo Horizonte, Porto Alegre and Vitória.^3^ It has investigated the association between the subjects’ self-perception of the opportunities for physical activity in their neighborhoods (by applying the “walking environment” scales that were originally used in the Multi-Ethnic Study of Atherosclerosis) and their frequency of leisure-time physical activity (LTPA), through the International Physical Activity Questionnaire. The result was that perception that the neighborhood was more walkable was positively associated with engaging in LTPA and doing so for longer periods per week. Compared with subjects who saw their community as less walkable, those who perceived it as more walkable had a 70% greater chance of engaging in LTPA.^6^ The favorable effects of LTPA were shown to be a 22% lower possibility of a coronary event for active women than for inactive women and 33% lower for active men than for inactive men, according to the 10-year Framingham Risk Score.^7^

Among the female participants of ELSA-Brasil, there was a higher frequency of hypertension among those who were physically active during their journey to work than among those who were inactive.^8^ These associations were maintained after adjustment for LTPA and socioeconomic variables. One speculation to explain the high prevalence of hypertension among individuals who were active during their journey to work in ELSA-Brasil could be their greater exposure to air pollution from traffic, as described in China^9^^,^^10^ and the United States.^11^^,^^12^^,^^13^ This has also been described among urban roadway law enforcement officers in Brazil.^14^

In conclusion, location matters because it provides the walkability conditions for leisure-time physical activity, but walking to go to work might be deleterious in Brazilian cities, perhaps because this increases the exposure to air pollutants.

## THE REALITY OF SÃO PAULO, BRAZIL

Despite an overall decline in cardiovascular mortality rates in São Paulo, the downward trends have been slower in the poorest areas than in the wealthiest ones, thus widening the social gap regarding these diseases.^1^^,^^2^ São Paulo is the tenth most populated city in the world, with 96 districts spread over a large area. Working-class people live in neighborhoods that are far from the work sites, and this implies a mean commuting time of longer than two hours per day. Most of them commute by bus, which accounts for 47% of the kilometers traveled, while private motor vehicles account for 29.5%, subway (metro) for 12.8%, walking or cycling for 7% and motorcycles for 4%.^15^ Ninety-five percent of the buses have diesel engines that produce exhaust containing combustion-derived particulate matter < 2.5 mm (PM_2.5_). Consequently, the air quality in São Paulo is considered unhealthy during all seasons, with reports of PM_2.5_ concentrations reaching 750 mg/m^3^ (30 times the daily threshold for hazardous levels).^16^ Moreover, most of these districts have few or no parks for exercise or cultural activities. Particulate material is associated with incidence of heart diseases and mortality due to these diseases.^17^ PM_2.5_ leads to increased oxidative stress associated with endothelial dysfunction and, consequently, dysregulation of the autonomic nervous system, which is a putative pathway for high blood pressure.^18^

## A PROPOSAL FOR THE MAYOR AND THE CITY COUNCIL OF SÃO PAULO TO REDUCE CARDIOVASCULAR DEATHS

According to data from ELSA-Brasil and several other studies, two important measures can be proposed to the municipality to improve cardiovascular health. Firstly, creation and expansion of the number of green areas in the less affluent areas. This action may be effective for improving cardiovascular health, as demonstrated through the Nurses’ Health Study results, which showed that higher levels of green vegetation were associated with decreased cardiovascular mortality.^19^

Secondly, since cardiovascular events and deaths have been strongly correlated with PM_2.5_, and buses are the largest source of these pollutants, we are giving our support to the new bill of law that is currently under discussion in the City Council to progressively restrict the number of buses fueled by diesel until they have been totally replaced by cleaner vehicles.

Please, Mr. Doria, give up your marketing-driven policies and adopt science-driven actions to reduce the burden of cardiovascular diseases.
